# SCREENING OF US VETERANS AT HIGH RISK FOR OUTCOMES FOLLOWING COVID-19 BY FRAILTY STATUS

**DOI:** 10.1093/geroni/igac059.2114

**Published:** 2022-12-20

**Authors:** Catherine Park, Aanand Naik, Molly Horstman, Benjamin Seligman, Ariela Orkaby, Orna Intrator, Kristine Lynch, Javad Razjouyan

**Affiliations:** Veterans Affairs, Houston, Texas, United States; Veterans Affairs, Houston, Texas, United States; Veterans Affairs, Houston, Texas, United States; David Geffen School of Medicine at UCLA, Los Angeles, California, United States; VA Boston, Boston, Massachusetts, United States; Canandaigua VA Medical Center, Canandaigua, New York, United States; University of Utah, Salt Lake City, Utah, United States; Veterans Affairs, Houston, Texas, United States

## Abstract

Frailty increases risk of adverse outcomes in the presence of stressors such as COVID-19 infection. We examined the association between different levels of frailty and outcomes following COVID-19 infection. This is a retrospective cohort study of US Veterans aged ≥50 years, active Veterans Health Administration (VHA) care users, and tested positive for COVID-19 between 02/15/2020 and 09/30/2021. VHA frailty index (VA-FI) was calculated from one year prior to the COVID-19 testing and divided into three groups: robust (≤0.1), prefrail (0.1-0.2), and frail (>0.2). The risk of hospitalization, ICU admission, ventilator use, and in-hospital mortality were calculated using logistic regression adjusted by age, sex, body-mass-index, and race. The performance of VA-FI in predicting outcomes was compared to age or Charlson comorbidity index (CCI) using area under the curve (AUC). Of 204,426 COVID-19 positive Veterans, 32,965 were hospitalized (age: 71.4±10.4 years, BMI: 29.5±7.1 kg/m2). We observed higher odds of hospitalization (frail, adjusted odds ratio (aOR)=8.64; prefrail, aOR=2.57), ICU admission (frail, aOR=1.58; prefrail, aOR=1.32), ventilator use (frail, aOR=1.97; prefrail, aOR=1.57), and mortality (frail aOR=2.15; prefrail, aOR=1.55) in frail and prefrail compared to robust Veterans. We observed that VA-FI had higher AUC in predicting hospitalization (AUC 0.75) independent of age (0.59) and CCI (0.63). Veterans with COVID-19 who were frail and prefrail had a higher risk of hospitalization, ICU admission, ventilator use, mortality compared to robust. VA-FI may be a useful tool at the time of COVID-19 diagnosis to triage patients at risk for adverse outcomes.

